# Validation of risk prediction models for the development of HBV-related HCC: a retrospective multi-center 10-year follow-up cohort study

**DOI:** 10.18632/oncotarget.22375

**Published:** 2017-11-03

**Authors:** Yeon Seok Seo, Byoung Kuk Jang, Soon Ho Um, Jae Seok Hwang, Kwang-Hyub Han, Sang Gyune Kim, Kwan Sik Lee, Seung Up Kim, Young Seok Kim, Jung Il Lee

**Affiliations:** ^1^ Department of Internal Medicine, Korea University, Seoul, Korea; ^2^ Department of Internal Medicine, Keimyung University School of Medicine, Daegu, Korea; ^3^ Department of Internal Medicine, Institute of Gastroenterology, Yonsei University College of Medicine, Seoul, Korea; ^4^ Department of Internal Medicine, Soonchunhyang University Hospital, Bucheon, Korea

**Keywords:** hepatitis B, hepatocellular carcinoma, transient elastography, risk prediction, liver stiffness

## Abstract

Recently, modified REACH-B (mREACH-B) risk prediction model for hepatocellular carcinoma (HCC) development was proposed. We validated the accuracy of the mREACH-B model and compared its accuracy with those of other prediction models. Between 2006 and 2012, 1,241 patients with chronic hepatitis B (CHB) were recruited. All patients underwent transient elastography at enrollment. The median age of the study population (840 males, 401 females) was 49 years. The median PAGE-B, LSM-HCC, and mREACH-B values were 10, 10, and 8, respectively. Among patients without cirrhosis (*n* = 940, 75.7%), the median REACH-B value was 9. During the follow-up period (median 77.4 months), 66 (5.3%) and 83 (6.7%) patients developed HCC and liver-related events (LRE), respectively. Higher liver stiffness (LS) independently predicted HCC (hazard ratio [HR] = 1.047) and LRE development (HR = 1.047) (all *P* < 0.05). The mREACH-B significantly predicted HCC (AUC = 0.824 at 3-year and 0.750 at 5-year) and LRE development (AUC = 0.782 at 3-year and 0.739 at 5-year) (all *P* < 0.001) and it performed similarly or significantly better than the PAGE-B and LSM-HCC (AUC = 0.715-0.809 at 3-year and 0.719-0.742 at 5-year for HCC; AUC = 0.704-0.777 at 3-year and 0.721-0.735 at 5-year for LRE). Among patients without cirrhosis, mREACH-B predicted HCC (AUC = 0.803 vs. 0.654-0.816 at 3-year and 0.684 vs. 0.639-0.738 at 5-year) and LRE development (AUC = 0.734 vs. 0.619-0.789 at 3-year and 0.674 vs. 0.626-0.729 at 5-year) similarly to PAGE-B, REACH-B, and LSM-HCC. mREACH-B appropriately predicted HCC and LRE development in patients with CHB and showed similar or superior accuracy to those of PAGE-B, REACH-B, and LSM-HCC.

## INTRODUCTION

Chronic hepatitis B (CHB) virus infection is a leading cause of liver cirrhosis and hepatocellular carcinoma (HCC), especially in Asian countries, where chronic hepatitis B virus (HBV) infection is endemic [1, 2]. If HCC is diagnosed at an early stage during surveillance, the chance of a ‘cure’ and corresponding favorable long-term outcomes can be expected. Thus, risk stratification and the early detection of HCC are of great importance in patients with CHB.

To date, several risk prediction models including the REACH-B and PAGE-B models showing acceptable accuracy have been proposed [3]. Of these, the REACH-B model, which includes gender, age, alanine aminotransferase (ALT) level, hepatitis B e antigen (HBeAg) status, and HBV DNA level as variables, was proposed from an Asian multi-center study [3]. However, because REACH-B was established from the cohort of CHB patients without cirrhosis, it is not applicable to the whole spectrum of patients with CHB, such as those with cirrhosis, who are at higher risk of HCC development and may benefit most from risk stratification. In addition, the prognostic accuracy of the PAGE-B model, which was established from Caucasian subjects with CHB [4], has not fully validated yet in Asian subjects.

Recently, based on the concept that detailed stratification according to fibrotic burden can be more prognostic [5], several liver stiffness (LS)-based risk prediction models such as the modified REACH-B (mREACH-B) and LSM-HCC models have been proposed. Although the prognostic accuracy of mREACH-B [6], which was established from REACH-B model by incorporating LS, assessed using transient elastography (TE) instead of HBV DNA, has been confirmed in several studies [7, 8], its external validation has yet to be performed. In addition, LSM-HCC model was recently proposed [9]. However, further validation of its prognostic accuracy and the comparison with mREACH-B are still required.

Thus, in this multi-center retrospective cohort study, we sought to validate the prognostic accuracy of mREACH-B in predicting the risk of HCC and liver-related event (LRE) development in comparison with other risk prediction models, including PAGE-B, REACH-B, and LSM-HCC models, in patients with CHB.

## MATERIALS AND METHODS

### Patients

A total of 1,538 patients with CHB who underwent TE examinations at four tertiary institutions (Gangnam Severance Hospital, Dongsan Medical Center, Korea University Hospital, and Soonchunhyang University Bucheon Hospital) from 2006 to 2012 were recruited for this retrospective multi-center cohort study. CHB was defined as the persistent presence of the serum HBV surface antigen for > 6 months.

Exclusion criteria were as follows: 1) TE examination failure (valid shot = 0), 2) unreliable LS values, 3) HCC or LRE development < 6 months after enrollment, 4) current or previous history of HCC, decompensation, or liver transplantation, 5) Child-Pugh class B or C, 6) co-infection with hepatitis C or HIV, 7) right-sided heart failure, 8) ascites or pregnancy, 9) significant alcohol consumption (> 40 g/daily), 10) significant medical comorbidities, and 11) insufficient data for risk model calculation (Supplementary Figure 1).

The study conformed to the ethical guidelines of the 1975 Declaration of Helsinki and was approved by the institutional review board of each institute. The requirement for written informed consent was waived due to the retrospective nature of the study.

### Follow-up

Each patient was screened for HCC with ultrasonography at their initial visit. If no evidence of HCC was detected, patients were followed up with αfetoprotein and ultrasonography every 3 or 6 months for HCC surveillance. Antiviral therapy (AVT) was started according to the guidelines of the Korean Association for the Study of the Liver [10]. During surveillance, HCC was diagnosed based on the guidelines of the American Association for the Study of Liver Diseases [1].

### Primary end-points

The primary aim of this study was to evaluate the predictive value of risk prediction models for assessing the risks of HCC and LRE development. To avoid statistical repetition, we selected the earliest of LREs as a major event if a given patient experienced different types of LRE at different times. LRE included the development of HCC, decompensation, liver transplantation, and liver-related death.

### Assessment of liver stiffness using transient elastography

The LS value was assessed using TE (FibroScan; EchoSens, Paris, France). LS values measured by experienced technicians or nurses (> 500 examinations) were expressed in kilopascals (kPa). The detailed process for TE assessment has been described previously [11–13]. The interquartile range (IQR) served as an index of the intrinsic variability in LS values and corresponded to the interval of LS results containing 50% of the valid measurements between the 25th and 75th percentiles. The median value of the successful measurements was regarded as representative of the LS value only if the IQR to median value ratio was < 30%. In addition, the LS value with at least 10 valid measurements and a success rate > 60% was considered reliable.

### Selection of risk prediction models for comparison

First, the REACH-B model, from which mREACH-B was derived, was selected [14]. The mREACH-B score substitutes the LS value for the HBV DNA level in the REACH-B model [6]. Additionally, another LS-based LSM-HCC model was selected to compare its prognostic accuracy with mREACH-B [9]. The LSM-HCC score was generated from the LS values, age, serum albumin level, and HBV DNA level. Finally, we selected a recently proposed prediction model from Caucasian patients with CHB receiving AVT [15], named PAGE-B, which has age, gender, and platelet count, as constituent variables, because only one previous study validated PAGE-B in Asian patients with CHB with no comparison with the accuracy of LS-based models [4]. The detailed calculation methods are summarized in Supplementary Table 1.

### Statistical analysis

Data are expressed as medians with IQRs or as *n* (%), as appropriate. Student's *t*-test (or the Mann-Whitney test) and the χ^2^ test (or Fisher's exact test) were used to compare the baseline characteristics of patients with and without liver cirrhosis at baseline, which was diagnosed based on ultrasonographic findings, including splenomegaly, blunt angle, and morphological changes (nodularity of liver surface, atrophy of the right lobe, hypertrophy of the left and caudate lobes, expansion of periportal spaces, and intrahepatic nodules). For subgroup analysis, high ALT level was defined as > 40 IU/mL. Additionally, baseline characteristics of patients who developed HCC or LRE and those who did not were compared. Patients were censored at the time of first presentation of HCC or LRE according to the selection of end-points or at the last follow-up. The annual and cumulative incidence rates of HCC were calculated using the Kaplan-Meier method. To identify independent risk factors for HCC development, univariate and subsequent multivariate Cox proportional hazard regression analyses were conducted. Hazard ratios (HRs) and corresponding 95% confidence intervals (CIs) are presented. The 3-, 5-, and 7-year cumulative incidences of HCC were assessed by calculating area under the curves (AUCs). The AUCs of the risk prediction models were compared between pairs using the method of Delong *et al*. A *P* value *<* 0.05 (two-tailed test) was considered to indicate statistical significance. All analyses were performed using the SPSS software (ver. 20.0; SPSS Inc., Chicago, IL, USA).

## RESULTS

### Baseline characteristics

After excluding 61 patients due to TE examination failure or unreliable LS values (drop-out rate due to TE, 4.0%), 1,477 patients with a reliable LS value for calculating risk prediction models were selected. Then, a further 236 patients were excluded according to our exclusion criteria. Finally, 1,241 patients with CHB were included in this retrospective multi-center cohort study (Supplementary Figure 1).

Baseline characteristics at enrollment are summarized in Table 1. The median age of the patients (840 males, 401 females) was 49 years. In total, 301 (24.3%) patients had liver cirrhosis and 557 (44.9%) were receiving AVT at enrollment. The median LS value was 3.9 kPa. Additionally, the median PAGE-B value was 10 and those of the LS-based prediction models of LSM-HCC and mREACH-B were 10 and 8, respectively. Among patients without liver cirrhosis, the median REACH-B value was 9.

**Table 1:** Baseline characteristics of the study population (*n* = 1,241)

Variables	All	Liver cirrhosis		
		Without (940, 75.7%)	With (301, 24.3%)	*P* value
**Demographic variables**				
Age, years	49 (40–57)	47 (38–55)	54 (48–61)	< 0.001
Male gender	840 (67.7)	617 (65.6)	223 (74.1)	0.007
BMI, kg/m^2^	23.5 (21.5–25.4)	23.3 (21.4–25.4)	23.8 (22.0–25.5)	0.141
Diabetes	92 (7.4)	55 (5.9)	37 (12.3)	0.001
Hypertension	109 (8.8)	75 (8.0)	34 (11.3)	0.080
On-going AVT	557 (44.9)	393 (41.8)	164 (54.5)	< 0.001
**Laboratory variables**				
Total bilirubin, mg/dL	0.8 (0.6–1.0)	0.8 (0.6–1.0)	0.9 (0.7–1.1)	< 0.001
Serum albumin, g/dL	4.4 (4.1–4.6)	4.4 (4.1–4.6)	4.3 (4.1–4.5)	< 0.001
AST, IU/L	32 (23–53)	30 (22–52)	36 (27–53)	0.004
ALT, IU/L	35 (22–71)	35 (21–79)	35 (24–56)	< 0.001
AFP, ng/mL	3.10 (2.10–5.00)	2.90 (2.00–4.50)	3.95 (2.55–7.00)	0.537
HBeAg positivity	562 (45.3)	452 (48.1)	110 (36.5)	0.001
HBV DNA, log_10_ IU/mL	3.9 (2.0–6.6)	4.0 (2.1–7.0)	3.6 (1.6–5.8)	0.001
Platelet count, 10^9^/L	185 (142–228)	198 (161–237)	136 (101–179)	< 0.001
**Liver stiffness, kPa**	3.9 (4.7–11.0)	6.1 (4.5–8.7)	11.9 (7.9–17.4)	< 0.001
**Risk prediction models**				
PAGE-B	10 (8–15)	9 (7–14)	14 (10–16)	< 0.001
REACH-B	-	9 (7–11)	-	-
LSM-HCC	10 (5–18)	10 (0–15)	18 (10–24)	< 0.001
mREACH-B	8 (6–10)	7 (6–9)	10 (8–12)	< 0.001

Variables are expressed as median (interquartile range) or n (%).

BMI, body mass index; AVT, antiviral therapy; AST, aspartate aminotransferase; ALT, alanine aminotransferase; AFP, alpha-fetoprotein; HBeAg, hepatitis B e antigen; kPa, kilopascal.

### Comparison between patients with and without liver cirrhosis

When the study population was stratified into two groups, with and without liver cirrhosis (Table 1), age, the proportions of male gender, diabetes, and on-going AVT, total bilirubin level, and LS value were significantly higher in patients with liver cirrhosis than in those without it, whereas serum albumin level, aspartate aminotransferase (AST) level, ALT level, the proportion of HBeAg positivity, HBV DNA level, and platelet count were significantly lower in patients with liver cirrhosis (all *P* < 0.05). PAGE-B, LSM-HCC, and mREACH-B values were significantly higher in patients with liver cirrhosis than in those without it (all *P* < 0.001).

### HCC and LRE development

**Figure 1:**
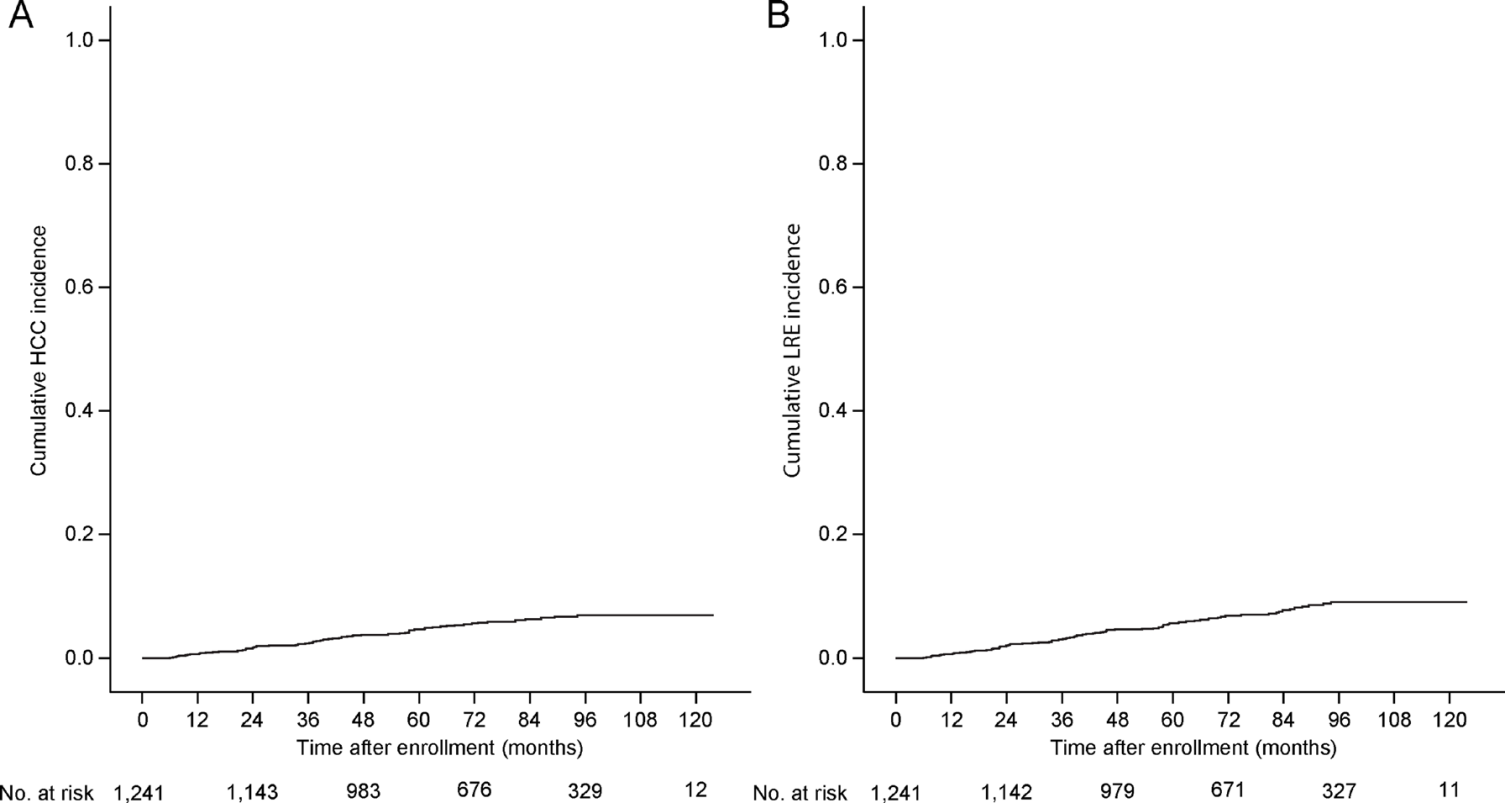
Cumulative incidence rates of HCC and LRE (Kaplan-Meier plot) The cumulative incidence rates of HCC at 3, 5, and 7 years were 2.4%, 4.6%, and 6.3%, respectively (**A**), whereas those of LRE were 3.0%, 5.6%, and 7.7%, respectively (**B**). HCC, hepatocellular carcinoma; LRE, liver-related event.

The median follow-up period from enrollment was 77.4 (IQR 56.2–97.7) months. In total, 66 (5.3%) and 83 (6.7%) patients developed HCC and LRE, respectively. For patients who developed LRE, HCC mostly comprised LRE (64, 77.1%), followed by decompensation (*n* = 17, 20.5%) and liver-related death (*n* = 2, 2.4%). The cumulative incidence rates at 3, 5, and 7 years were 2.4%, 4.6%, and 6.3% for HCC development and 3.0%, 5.6%, and 7.7% for LRE development, respectively (Figure 1).

When the study population was stratified according to liver cirrhosis, patients with liver cirrhosis experienced HCC and LRE development more frequently (1.1%, 2.6%, and 3.5% for HCC and 8.8%, 15.1%, and 20.6% for LRE, respectively) than those without it (0.7%, 2.0%, and 2.5% for HCC and 7.7%, 12.5%, and 17.8% for LRE, respectively; log-rank test, all *P* < 0.001; Supplementary Figure 2).

### Comparisons between patients who developed HCC and LRE and those who did not

Characteristics of the 66 (5.3%) patients who developed HCC and the 1,174 (94.6%) patients who did not and the 83 (6.7%) patients who developed LRE and the 1,158 (93.3%) patients who did not are shown in Table 2. The age and proportions of hypertension and liver cirrhosis, total bilirubin level, LS value, and the values of three prediction models (PAGE-B, LSM-HCC, and mREACH-B) were significantly higher in patients who developed HCC than in those who did not (all *P* < 0.05), whereas serum albumin level, ALT level, and platelet count were significantly lower in patients who developed HCC (all *P* < 0.05). Similar findings were noted between patients who developed LRE and those who did not.

**Table 2:** Baseline characteristics of patients who developed HCC and LRE versus those who did not

Variables	Hepatocellular carcinoma			Liver-related event		
	Without	With	*P* value	Without	With	*P* value
	(*n* = 1,174, 94.6%)	(*n* = 66, 5.3%)		(*n* = 1,158, 93.3%)	(*n* = 83, 6.7%)	
**Demographic variables**						
Age, years	49 (40–56)	58 (51–64)	< 0.001	49 (39–56)	57 (50–64)	< 0.001
Male gender	789 (67.2)	50 (75.8)	0.176	780 (67.4)	60 (72.3)	0.396
Body mass index, kg/m^2^	23.4 (21.4–25.4)	24.2 (22.8–25.5)	0.917	23.4 (21.5–25.4)	23.8 (22.0–25.4)	0.684
Diabetes	85 (7.2)	7 (10.6)	0.329	83 (7.2)	9 (10.8)	0.198
Hypertension	93 (7.9)	16 (24.2)	< 0.001	89 (7.7)	20 (24.1)	< 0.001
Liver cirrhosis	255 (21.7)	46 (69.7)	< 0.001	246 (21.2)	55 (66.3)	< 0.001
On-going AVT	522 (44.5)	34 (51.5)	0.309	520 (44.9)	37 (44.6)	0.999
**Laboratory variables**						
Total bilirubin, mg/dL	0.8 (0.6–1.0)	0.9 (0.6–1.2)	0.049	0.8 (0.6–1.0)	0.9 (0.6–1.1)	0.035
Serum albumin, g/dL	4.4 (4.1–4.6)	4.2 (4.0–4.5)	0.002	4.4 (4.1–4.6)	4.2 (4.0–4.5)	0.002
Aspartate aminotransferase, IU/L	31 (23–52)	45 (32–62)	0.146	31 (23–52)	44 (19–63)	0.211
Alanine aminotransferase, IU/L	35 (21–71)	40 (28–63)	0.001	35 (22–71)	40 (25–64)	0.001
Alpha-fetoprotein, ng/mL	3.10 (2.10–4.88)	4.70 (2.50–10.15)	0.749	3.10 (2.10–6.70)	4.10 (2.33–9.43)	0.704
HBeAg positivity	536 (45.7)	26 (39.4)	0.374	528 (45.6)	34 (41.0)	0.427
HBV DNA, log IU/mL	3.8 (2.0–6.7)	5.3 (2.3–6.5)	0.623	3.9 (2.1–6.7)	3.8 (1.7–6.4)	0.596
Platelet count, 10^9^/L	190 (144–229)	125 (1034–170)	< 0.001	190 (145–229)	124 (90–177)	< 0.001
Liver stiffness, kPa	6.8 (4.8–10.5)	12.2 (8.4–21.2)	< 0.001	6.8 (4.8–10.4)	12.0 (7.9–20.9)	< 0.001
Risk prediction models						
PAGE-B	10 (7–15)	15 (11–16)	< 0.001	10 (7–15)	15 (10–16)	< 0.001
LSM-HCC	10 (5–18)	19 (14–24)	< 0.001	10 (5–18)	18 (10–24)	< 0.001
mREACH-B	8 (6–10)	11 (9–13)	< 0.001	8 (6–10)	11 (9–13)	< 0.001

Variables are expressed as median (interquartile range) or n (%).

HCC, hepatocellular carcinoma; LRE, liver-related event; AVT, antiviral therapy; HBeAg, hepatitis B e antigen; kPa, kilopascal.

### Independent predictors of HCC and LRE development

Independent predictors of HCC and LRE development were evaluated (Table 3). Univariate analyses identified age, liver cirrhosis, total bilirubin, serum albumin, platelet count, and LS value as significant predictors of HCC development, whereas age, liver cirrhosis, serum albumin, platelet count, and LS value were selected as significant predictors of LRE development (all *P* < 0.05). According to a multivariate analysis, older age (HR = 1.065), liver cirrhosis (HR = 2.724), and higher LS value (HR = 1.047) were independently associated with an increased risk of HCC development (all *P* < 0.05), whereas older age (HR = 1.057), liver cirrhosis (HR = 2.299), lower platelet count (HR = 0.994), and higher LS value (HR = 1.047) were independently associated with an increased risk of LRE development (all *P* < 0.05).

**Table 3:** Independent predictor of HCC and LRE development

Variables	Hepatocellular carcinoma				Liver-related event			
	Univariate	Multivariate			Univariate	Multivariate		
	*P* value	Hazard ratio	95% CI	*P* value	*P* value	Hazard ratio	95% CI	*P* value
**Demographic variables**								
Age, years	< 0.001	1.065	1.038–1.093	< 0.001	< 0.001	1.057	1.034–1.081	<0.001
Male gender	0.173	-	-	-	0.398			
Body mass index, kg/m^2^	0.853	-	-	-	0.725			
Diabetes	0.335	-	-	-	0.230			
Liver cirrhosis	< 0.001	2.724	1.495–4.965	0.001	< 0.001	2.299	1.362–3.881	0.002
On-going AVT	0.335	-	-	-	0.798			
**Laboratory variables**								
Total bilirubin, mg/dL	0.021	1.369	0.768–2.437	0.287	0.052			
Serum albumin, g/dL	0.001	1.054	0.484–2.295	0.895	< 0.001	1.135	0.567–2.271	0.720
Aspartate aminotransferase, IU/L	0.582	-	-	-	0.999			
Alanine aminotransferase, IU/L	0.163	-	-	-	0.140			
Alpha-fetoprotein, ng/mL	0.733	-	-	-	0.650			
HBeAg positivity	0.269	-	-	-	0.351			
HBV DNA, log IU/mL	0.693	-	-	-	0.508			
Platelet count, 109/L	< 0.001	0.996	0.991–1.001	0.144	< 0.001	0.994	0.989–0.999	0.012
**Liver stiffness, kPa**	< 0.001	1.047	1.018–1.077	0.002	< 0.001	1.047	1.021–1.074	< 0.001

HCC, hepatocellular carcinoma; LRE, liver-related event; CI, confidence interval; AVT, antiviral therapy; HBeAg, hepatitis B e antigen; kPa, kilopascal.

### Unadjusted hazard ratio of models in predicting HCC and LRE development

The unadjusted HRs obtained from the prediction models are shown in Table 4. PAGE-B, LSM-HCC, and mREACH-B were significantly predictive of HCC and LRE development (HR = 1.176–1.366 for HCC and 1.109–1.325 for LRE; all *P* < 0.001). In patients without liver cirrhosis, PAGE-B, REACH-B, LSM-HCC, and mREACH-B were predictive of HCC and LRE development (HR=1.116–1.246 for HCC and 1.087–1.214 for LRE; all *P* < 0.05), except for REACH-B in predicting LRE (*P* = 0.115). In patients with liver cirrhosis, PAGE-B, LSM-HCC, and mREACH-B were also predictive of HCC and LRE development (HR = 1.074–1.248 for HCC and 1.067–1.218 for LRE; all *P* < 0.05) except PAGE-B in predicting HCC (*P* = 0.100).

**Table 4:** Unadjusted hazard ratios of prediction models

Models	Hepatocellular carcinoma			Liver-related event		
	Hazard ratio	95% CI	*P* value	Hazard ratio	95% CI	*P* value
All						
PAGE-B	1.176	1.107–1.250	< 0.001	1.158	1.098–1.220	< 0.001
REACH-B	-	-	-	-	-	-
LSM-HCC	1.125	1.091–1.160	< 0.001	1.109	1.080–1.138	< 0.001
mREACH-B	1.366	1.256–1.486	< 0.001	1.325	1.231–1.426	< 0.001
Without cirrhosis						
PAGE-B	1.174	1.057–1.303	0.003	1.104	1.017–1.198	0.018
REACH-B	1.219	1.048–1.417	0.010	1.103	0.976–1.247	0.115
LSM-HCC	1.116	1.057–1.178	< 0.001	1.087	1.039–1.137	< 0.001
mREACH-B	1.246	1.074–1.445	0.004	1.214	1.071–1.376	0.002
With cirrhosis						
PAGE-B	1.069	0.987–1.158	0.100	1.090	1.011–1.176	0.026
REACH-B	-	-	-	-	-	-
LSM-HCC	1.074	1.033–1.117	< 0.001	1.067	1.030–1.105	< 0.001
mREACH-B	1.248	1.113–1.399	< 0.001	1.218	1.101–1.349	< 0.001

CI, confidence interval.

### Predictive accuracy of models in predicting HCC and LRE development

**Figure 2:**
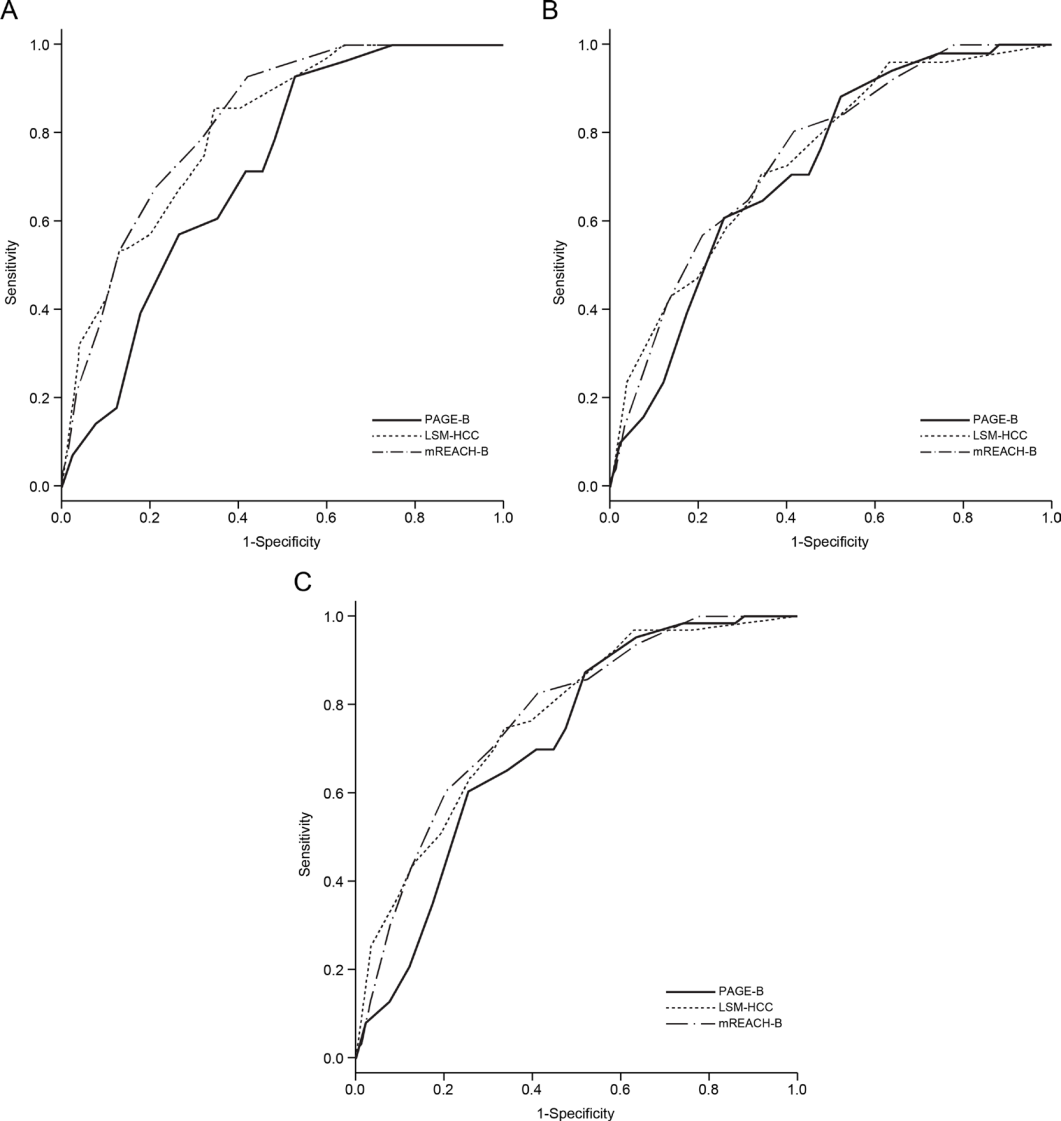
Receiver operating characteristic curves of the PAGE-B, LSM-HCC, and mREACH-B scores for the prediction of HCC development at 3 (**A**), 5 (**B**), and 7 years (**C**) in the entire study population. HCC, hepatocellular carcinoma.

The performance of the risk prediction models in predicting HCC at 3, 5, and 7 years was calculated (Table 5, Figure 2). The accuracy of REACH-B was calculated only for patients without liver cirrhosis.

**Table 5:** Comparison of the prognostic accuracy of risk prediction models for HCC development

Study groups	At 3 years		At 5 years			At 7 years			
Prediction models	AUC	95% CI	*P* value^1^	AUC	95% CI	*P* value^1^	AUC	95% CI	*P* value^1^
All (*n =* 1,241)									
PAGE-B	0.715^**^	0.644–0.787	0.014	0.719^**^	0.659–0.779	0.445	0.714^**^	0.661–0.766	0.111
REACH-B	-	-	-	-	-	-	-	-	-
LSM-HCC	0.809^**^	0.742–0.876	0.444	0.742^**^	0.677–0.809	0.639	0.765^**^	0.709–0.821	0.781
mREACH-B	0.824^**^	0.765–0.884	-	0.750^**^	0.689–0.811	-	0.770^**^	0.717–0.823	-
With normal ALT (*n =* 707)									
PAGE-B	0.734^*^	0.652–0.817	0.174	0.704^**^	0.630–0.778	0.541	0.699^**^	0.628–0.770	0.541
REACH-B	-	-	-	-	-	-	-	-	-
LSM-HCC	0.793^**^	0.652–0.884	0.312	0.742^**^	0.663–0.822	0.842	0.740^**^	0.665–0.816	0.687
mREACH-B	0.823^**^	0.740–0.906	-	0.737^**^	0.658–0.816	-	0.730^**^	0.654–0.807	-
With high ALT (*n =* 534)									
PAGE-B	0.696^*^	0.577–0.814	0.004	0.737^**^	0.638–0.836	0.359	0.736^**^	0.658–0.813	0.049
REACH-B	-	-	-	-	-	-	-	-	-
LSM-HCC	0.837^**^	0.744–0.929	0.658	0.766^**^	0.660–0.872	0.401	0.801^**^	0.723–0.879	0.447
mREACH-B	0.826^**^	0.744–0.907	-	0.786^**^	0.701–0.871	-	0.818^**^	0.755–0.880	-
Without cirrhosis (*n =* 940)									
PAGE-B	0.693	0.542–0.843	0.399	0.738^*^	0.648–0.829	0.535	0.721^*^	0.637–0.805	0.879
REACH-B	0.654	0.477–0.831	0.280	0.639	0.506–0.773	0.600	0.653^*^	0.535–0.772	0.221
LSM-HCC	0.816^*^	0.704–0.928	0.734	0.706^*^	0.577–0.836	0.556	0.725^*^	0.609–0.840	0.507
mREACH-B	0.803^*^	0.667–0.940	-	0.684^*^	0.564–0.804	-	0.691^*^	0.584–0.798	-
With cirrhosis (*n =* 301)									
PAGE-B	0.584	0.475–0.694	0.083	0.591	0.494–0.689	0.374	0.582	0.497–0.667	0.074
REACH-B	-	-	-	-	-	-	-	-	-
LSM-HCC	0.686^*^	0.574–0.798	0.643	0.630^*^	0.533–0.727	0.602	0.659^*^	0.575–0.743	0.483
mREACH-B	0.702^*^	0.598–0.805	-	0.644^*^	0.554–0.735	-	0.677^**^	0.598–0.755	-

^1^*P* value indicates the comparison between mREACH-B and other prediction models. AUC^*^ and ^**^ indicate *P* value of < 0.05 and < 0.001, respectively.

HCC, hepatocellular carcinoma; AUC, area under curve; CI, confidence interval; ALT, alanine aminotransferase.

In the entire study population, the mREACH-B score showed higher performance in predicting HCC than those of the PAGE-B and LSM-HCC at 3 years (AUC = 0.824 vs. 0.715 and 0.809, respectively), 5 years (AUC = 0.750 vs. 0.719 and 0.742, respectively), and 7 years (AUC = 0.770 vs. 0.714 and 0.765, respectively). However, a statistically significant difference was seen only between mREACH-B and PAGE-B at 3 years (*P* = 0.014).

When patients with normal ALT were selected (*n* = 707, 57.0%) to exclude the overestimating influence of high ALT on LS-based prediction models, AUC values of mREACH-B and LSM-HCC were higher than those of PAGE-B (AUC = 0.823–0.730 for mREACH-B, 0.793–0.740 for LSM-HCC vs. 0.734–0.699 for PAGE-B at 3–7 years), but the difference was not statistically significant (all *P* > 0.05). However, among patients with high ALT levels (*n* = 534, 43.0%), mREACH-B showed significantly higher AUC values than PAGE-B (0.826 vs. 0.696 at 3 years, *P* = 0.004, and 0.818 vs. 0.736 at 7 years, *P* = 0.049).

In the subgroup for patients without liver cirrhosis in whom REACH-B could be applied (*n* = 940, 75.7%), PAGE-B, LSM-HCC, and mREACH-B showed higher AUC values than REACH-B (AUC=0.738–0.693 for PAGE-B, 0.816–0.706 for LSM-HCC, and 0.803–0.684 for mREACH-B vs. 0.654–0.639 for REACH-B), but the difference was not statistically significant (all *P* > 0.05). Similar findings were seen in patients with liver cirrhosis (*n* = 301, 24.3%).

When the performance of the risk prediction models in predicting LRE at 3, 5, and 7 years was calculated (Supplementary Table 2, Supplementary Figure 3), overall trends for the predictive accuracy of prediction models were similar to those of HCC prediction.

### Predictive accuracy of models according to AVT status

When the patients with on-going AVT at enrollment were selected (*n* = 557), mREACH-B showed similar or higher AUC value than PAGE-B and LSM-HCC models at 3 years (0.846 vs. 0.674 and 0.809). At 5 and 7 years, AUC values of mREACH-B and LSM-HCC were similar (0.778 vs. 0.779 at 5 years; 0.788 vs. 0.789 at 7 years), but higher than those of PAGE-B model (0.688 at 5 years and 0.694 at 7 years). In addition, when the patients without on-going AVT were analyzed (*n* = 684), mREACH-B showed similar or higher AUC values than those of PAGE-B and LSM-HCC models (0.764 vs. 0.741 and 0.716 at 3 years; 0.735 vs. 0.734 and 0.735 at 5 years; 0.784 vs. 0.727 and 0.741 at 7 years).

### Risk stratification according to mREACH-B

We stratified the study population into four risk groups according to mREACH-B based on established cut-off values (Table 6) [8]. The cumulative incidence rates of HCC and LRE development were significantly different in low-risk versus low-intermediate-risk groups and between high-intermediate-risk and high-risk groups (all *P* < 0.05, log-rank test), whereas they were statistically similar between low-intermediate-risk and high-intermediate risk groups (*P* = 0.270, log-rank test; Supplementary Figure 4). Similar trends were noted with regard to LRE development (Table 6, Supplementary Figure 4). However, when high- and low-intermediate risk groups were merged as an intermediate-risk group, there were significantly different rates of HCC and LRE development (data not shown).

**Table 6:** Cumulative incidence rates of HCC and LRE development according to previously known cut-off values with mREACH-B

End-point Risk groups	3 years (%)	5 years (%)	7 years (%)	*P* value^1^
Hepatocellular carcinoma				
Low-risk < 6 (*n* = 262, 21.2%)	0	0	0	0.005
Low-intermediate-risk 6–7 (*n* = 306, 24.6%)	0.3	3.2	3.7	0.270
High-intermediate-risk 8–10 (*n* = 394, 31.7%)	2.1	3.9	4.7	< 0.001
High-risk >10 (*n* = 279, 22.5%)	7.2	11.6	17	-
Liver-related event				
Low-risk < 6 (*n* = 262, 21.2%)	0.4	0.4	0.4	0.005
Low-intermediate-risk 6–7 (*n* = 306, 24.6%)	1.0	3.9	4.4	0.152
High-intermediate-risk 8–10 (*n* = 394, 31.7%)	3.6	4.8	7.2	< 0.001
High-risk >10 (*n* = 279, 22.5%)	8.0	13.6	19.8	-

*P* value^1^ indicates the comparison with adjacent higher risk group.

HCC, hepatocellular carcinoma; LRE, liver-related event.

## DISCUSSION

An accurate assessment of the risk of HCC and LRE development is important for establishing individualized strategies of follow-up, intervention, and management, because this ultimately enables the extension of overall survival in patients with CHB. To address this, several risk prediction models for patients with CHB have been proposed [6, 9, 14, 15]. In this study, we attempted to validate the prognostic accuracy of the mREACH-B model, which is a modified version of the REACH-B model, and found that the mREACH-B model significantly predicted the risk of HCC and LRE development in the whole study population (AUC ≥ 0.750 for HCC and ≥ 0.739 for LRE at 3–7 years). Additionally, the mREACH-B model performed similarly or significantly better than the PAGE-B, REACH-B, and LSM-HCC models in our cohort.

Our study has several clinical implications. First, this study involved a large number of patients (*n* = 1,241) and a long follow-up period (median > 6 years and maximum around 10 years), which may have increased the statistical power and reliability. We attempted to obtain a larger number of patients who achieved our primary end-points during this long-term follow-up period (*n* = 66, 5.3% for HCC and *n* = 83, 6.7% for LRE). Additionally, this large sample size could facilitate stratification of the study population according to cirrhosis and ALT level. In the entire study population, the mREACH-B score showed significantly superior predictive value for HCC and LRE development at 3 years compared with that of PAGE-B (AUC = 0.824 vs. 0.715 for HCC and 0.782 vs. 0.704 for LRE, all *P* < 0.05), and similar predictive value to that of LSM-HCC at 3, 5, and 7 years. When a subgroup without cirrhosis was selected to investigate the accuracy of REACH-B, we found that the accuracy of mREACH-B was higher than that of the original REACH-B model (AUC = 0.803–0.684 vs. 0.639–0.654 for HCC and 0.734–0.674 vs. 0.626–0.600 for LRE). However, the difference was not statistically significant, probably due to the relatively small sample size of the participants and events in this subgroup without cirrhosis. Beyond these results, that the LS value was selected as an independent predictor of HCC and LRE development (both HR = 1.047) and REACH-B did not show a significant unadjusted HR for predicting LRE may support the superiority of LS-based risk prediction models. Additionally, stratification according to ALT level revealed that the accuracy of mREACH-B was maintained at 3–7 years (AUC = 0.723–0.792 in normal ALT vs. 0.786–0.756 in high ALT) and its similar or superior accuracy when compared to those of other models was also maintained, regardless of ALT level. In addition, in spite of well-known confounding influence of high ALT level on LS [16], the overall prognostic accuracy of LS-based risk models was slightly higher in the subgroup with high ALT level than that in the subgroup with normal ALT. Although the exact reasons are unclear, the relatively low ALT level of our study population (median 35 IU/L) and the high proportion of patients with on-going AVT at enrollment (around 45%) might have attenuated the influence of high ALT level. Thus, the influence of a high ALT level on LS-based risk prediction models should be investigated further [17].

Second, the mREACH-B model showed statistically similar accuracy in predicting HCC and LRE development to the LSM-HCC model in the entire study population and various subgroups, although previous studies showed the superior accuracy of mREACH-B [7, 8]. Because LSM-HCC included HBV DNA level, with diminished importance due to AVT [5], and mREACH-B was established based on the empirical weight allocation on LS stratification, the accuracy of LS-based prediction models may be altered according to the proportion of patients with on-going AVT and the different weight allocation strategy of the mREACH-B model for patients with high fibrotic burden. Further studies seem required to compare the predictive accuracy of these two LS-based risk models.

Third, we could compare the accuracy of PAGE-B with those of LS-based models. Although the PAGE-B model was recently proposed for Caucasian patients with CHB receiving AVT [15], only one validation study is currently available [4]; it concluded that the accuracy of PAGE-B was similar to that of the CU-HCC and GAG-HCC models, but significantly higher than that of the REACH-B model. In our study, AUC values of PAGE-B were also higher than those of REACH-B in predicting HCC and LRE development, but lower than those of LS-based models, although the differences were not statistically significant. Although the reason for this remains unclear, it may be explained in part by the platelet count, which is an important variable in the PAGE-B model, but was not selected as an independent predictor of HCC development (*P* = 0.144). Additionally, because the PAGE-B model was established from a cohort with AVT, its direct application in our cohort with mixed AVT status might have lessened the accuracy of the PAGE-B model.

Fourth, we tested not only the accuracy of risk prediction models for HCC development, but also for the comprehensive end-point of LRE, and found that all prediction models significantly predicted LRE development, except the REACH-B model (*P* = 0.115). Although HCC represented most LRE (77.1%), the AUC values of prediction models became slightly lower when LRE was used as an end-point. Because the PAGE-B, REACH-B, and LSM-HCC models were established to predict HCC development, further validation studies on the applicability of the models in predicting LRE should be conducted.

We are aware of several other issues that should be taken into consideration. First, the study population was derived retrospectively from tertiary academic institutions, which might have resulted in the relatively high proportion of patients with liver cirrhosis (24.4%). In addition, our definition of chronic hepatitis B might include HBV carriers rather than active hepatitis. Thus, our findings should be validated in future prospective studies involving the full spectrum of HBV disease in a community-based setting. Second, in some subgroups, the accuracy of the mREACH-B model was higher than those of other risk models. However, because the overall accuracy of the mREACH-B model in the entire population was not so prominent, we could not strongly insist the prognostic superiority of the mREACH-B score. Further validation studies are warranted to resolve this issue. Third, although all the technicians and nurses in our multi-center study who performed TE had sufficient experience [18], inter-institutional variability might have confounded our results. Additionally, the drop-out rates due to LS measurement failure or unreliable LS values (4.0%) were significantly lower than in a European study [19]. However, this failure rate seems similar to those of other Asian studies (from 1.1% to 3.5%) [20], probably due to the lower body mass index (median 23.5 kg/m^2^) in our study. Moreover, during TE examinations, the skin capsular distance was not measured. Because this thickness can influence the detection of advanced fibrosis by TE [21], future studies should take this issue into considerations. Fourth, the risks of HCC and LRE development can change, because clinical and laboratory variables, such as fibrotic burden (liver stiffness and liver cirrhosis), ALT levels, HBeAg status, and HBV DNA levels, can change due to prolonged AVT, especially in this era of potent antiviral agents. Thus, our next multi-center study would investigate the impact of AVT on risk prediction models and the optimal cutoffs which might help to triage patients into different surveillance strategies. Finally, a reduction in LS value during AVT has been reported to be a favorable prognostic factor [22]. Thus, dynamic risk assessment using the risk prediction models is important. However, because we focused first on validating the accuracy of mREACH-B, in comparison with other prediction models, based on baseline characteristics, the clinical implication of dynamic changes in risk prediction models was not investigated in this study. Further well-designed studies with serial assessments of risk prediction models, which can provide more relevant clinical information and validate our results, should be performed.

In conclusion, the mREACH-B model appropriately predicted HCC and LRE development in patients with CHB and showed similar or superior accuracy to PAGE-B, REACH-B, and LSM-HCC. However, further validation studies are needed to investigate whether to incorporate mREACH-B into current surveillance strategies and its feasibility in dynamic assessments in patients with CHB.

## SUPPLEMENTARY MATERIALS FIGURES AND TABLES



## Abbreviations

CHB: chronic hepatitis B
HCC: hepatocellular carcinoma
HBV: hepatitis B virus
AVT: antiviral therapy
AUC: area under curve
ALT: alanine aminotransferase
HBeAg: hepatitis B e antigen
mREACH-B: modified REACH-B
LS: liver stiffness
TE: transient elastography
LRE: liver-related event
HR: hazard ratio
CIs: confidence intervals
AST: aspartate aminotransferase
