# Supplementary material for: Validation of risk prediction models for the development of HBV-related HCC: a retrospective multi-center 10-year follow-up cohort study
Source: Oncotarget. 2017 Nov 3;8(68):113213–24. doi: 10.18632/oncotarget.22375 (PMC5762585; doi:10.18632/oncotarget.22375)
Supplement: Supplementary file 1 [file oncotarget-08-113213-s001.pdf]

Validation of risk prediction models for the development of HBV-related HCC: a retrospective multi-center 10-year follow-up cohort study

SUPPLEMENTARY MATERIALS

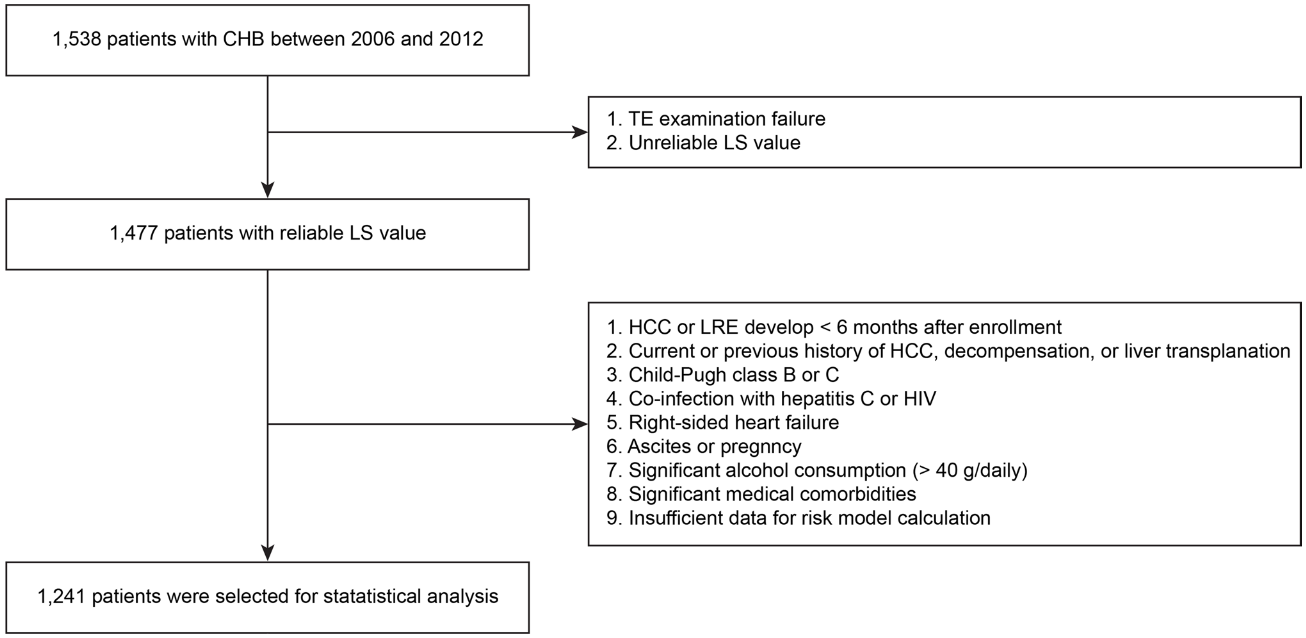

Supplementary Figure 1: Flow chart of study population selection. Among 1,538 patients with CHB, 1,477 with reliable LS values were enrolled, and after excluding 236 patients according to our exclusion criteria, 1,241 patients were included in the final statistical analysis. CHB, chronic hepatitis B; LS, liver stiffness; HCC, hepatocellular carcinoma; LRE, liver-related event.

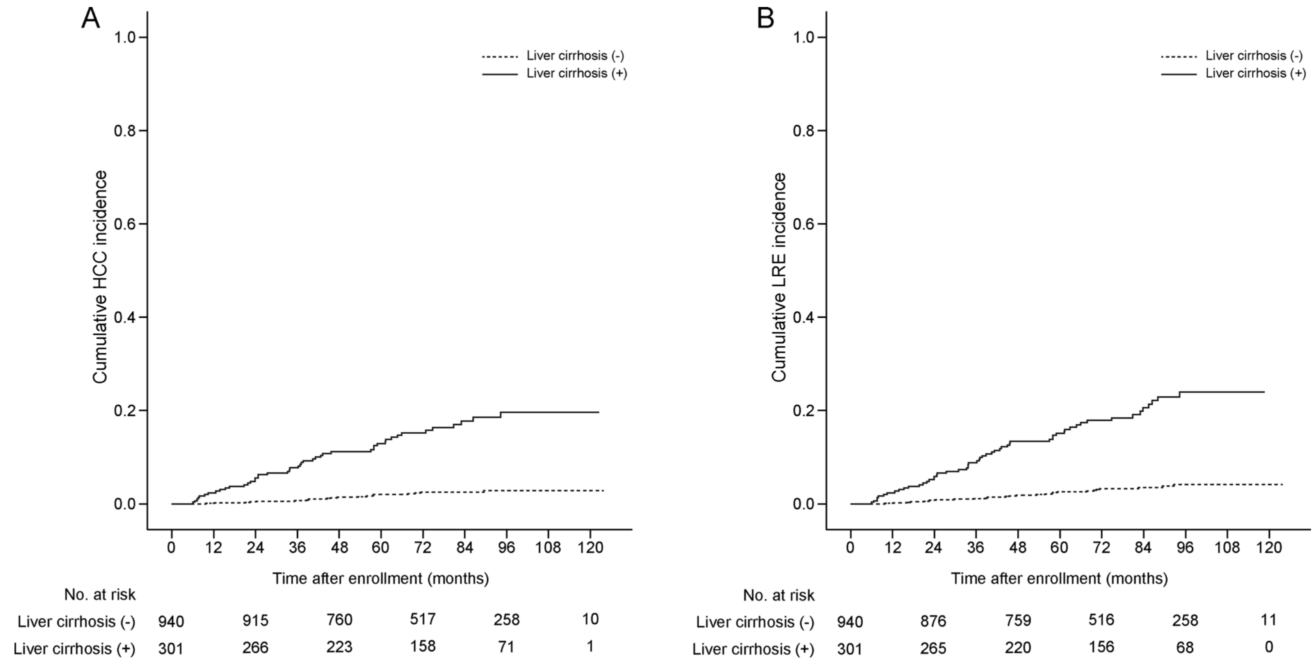

Supplementary Figure 2: Cumulative incidence rates of HCC and LRE according to the presence of liver cirrhosis (Kaplan-Meier plot). The cumulative incidence rates of HCC in patients without liver cirrhosis at 3, 5, and 7 years were 0.7%, 2.0%, and 2.5%, whereas those in patients with liver cirrhosis were 1.1%, 2.6%, and 3.5%, respectively (**A**). Additionally, the cumulative incidence rates of LRE in patients without liver cirrhosis at 3, 5, and 7 years were 7.7%, 12.9%, and 17.8.5%, whereas those in patients with liver cirrhosis were 8.8%, 15.1%, and 20.1%, respectively (**B**). HCC, hepatocellular carcinoma; LRE, liver-related event.

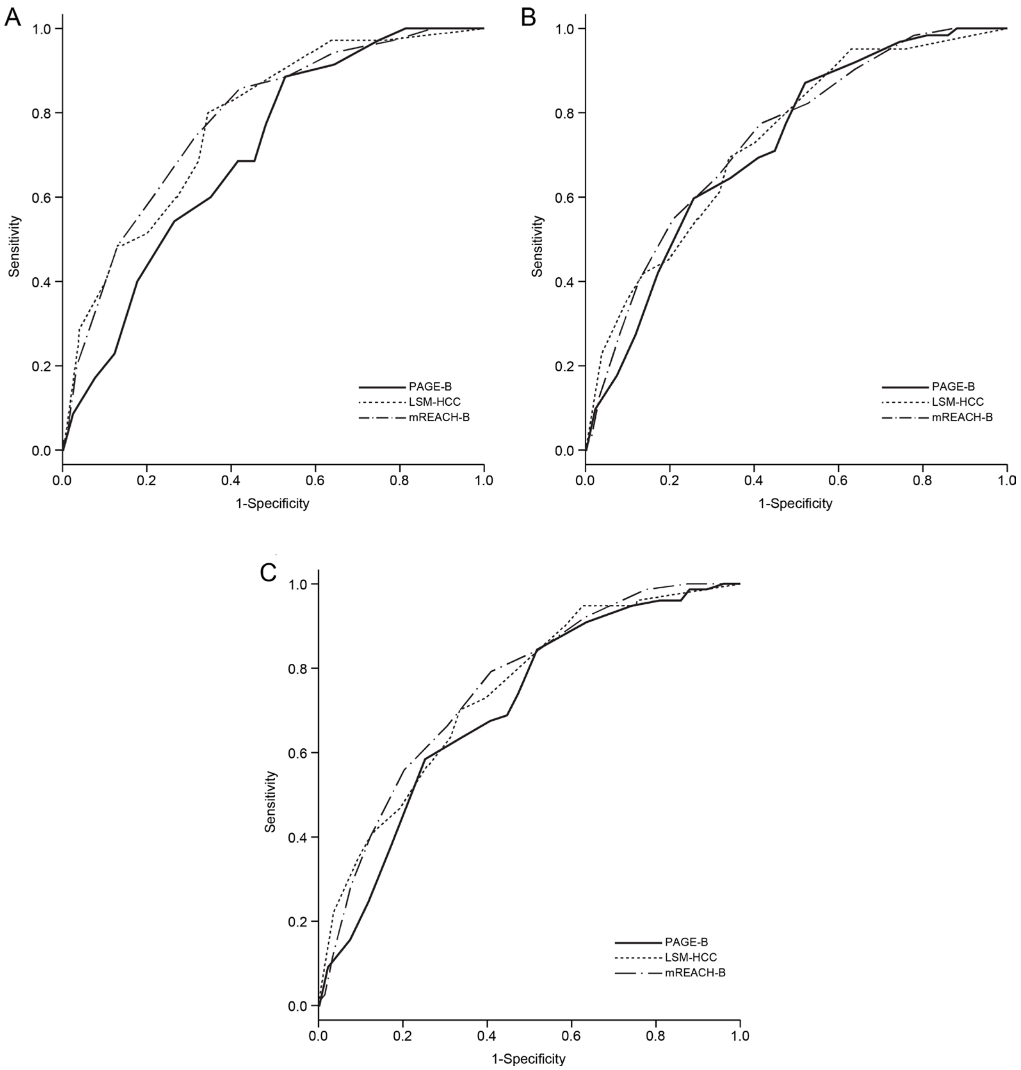

Supplementary Figure 3: Receiver operating characteristic curves of the PAGE-B, LSM-HCC, and mREACH-B scores for the prediction of LRE development at 3 (**A**), 5 (**B**), and 7 years (**C**) in the entire study population. LRE, liver-related event.

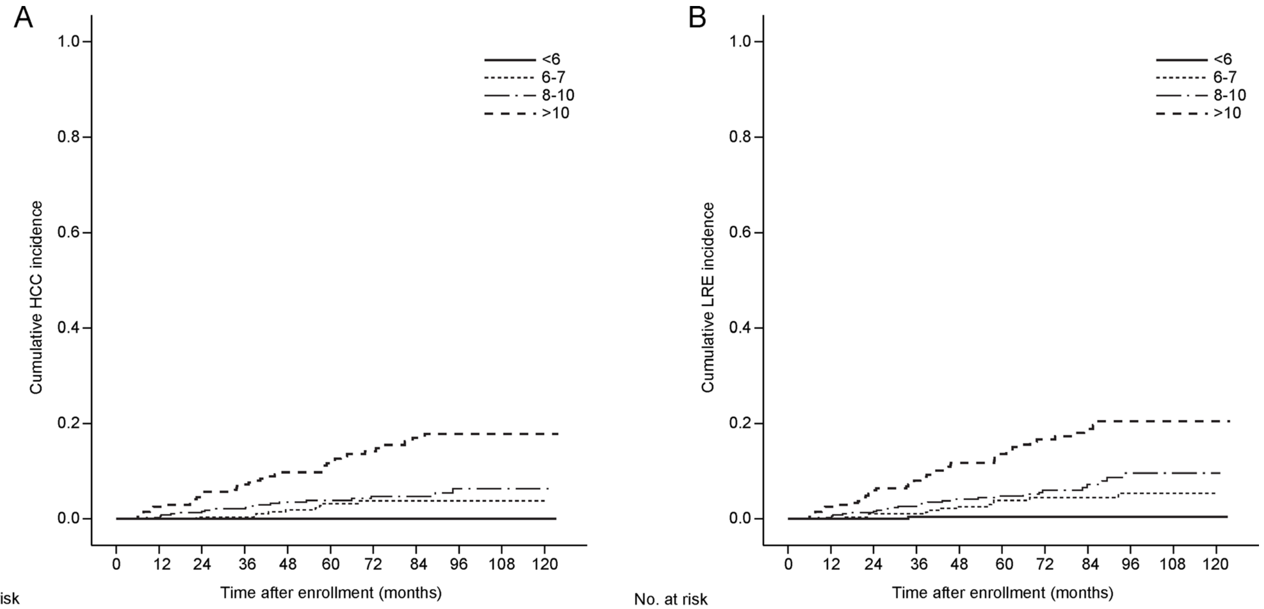

No. at risk						
mREACH-B<6	262	243	205	129	61	1
mREACH-B 6-7	306	285	247	169	87	2
mREACH-B 8-10	394	364	319	232	101	3
mREACH-B>10	279	248	208	142	80	3

No. at risk						
mREACH-B<6	262	243	205	129	57	1
mREACH-B 6-7	306	284	245	167	87	2
mREACH-B 8-10	394	364	319	232	100	3
mREACH-B>10	279	246	204	139	78	2

Supplementary Figure 4: Cumulative incidence rates of HCC (**A**) and LRE (**B**) according to the cut-off value**s** of mREACH-B (Kaplan-Meier plot). The cumulative incidence rates of HCC (A) and LRE development (B) were significantly different between the low-risk and low-intermediate-risk groups and between the high-intermediate-risk and high-risk groups (all $P < 0.05$, log-rank test), whereas they were similar between the low-intermediate-risk and high-intermediate risk groups ($P = 0.270$, log-rank test). Similar trends were noted in regard to LRE development. HCC, hepatocellular carcinoma; LRE, liver-related event.

Supplementary Table 1: Constituent variables and calculation methods of risk prediction

Variable	PAGE-B	REACH-B	mREACH-B
Age, years	16–29: 0	30–34: 0	30–34: 0
	30–39: 2	35–39: 1	35–39: 1
	40–49: 4	40–44: 2	40–44: 2
	50–59: 6	45–49: 3	45–49: 3
	60–69: 8	50–54: 4	50–54: 4
	≥70: 10	55–59: 5	55–59: 5
		60–65: 6	60–65: 6
Gender	Male: 6	Male: 2	Male: 2
	Female: 0	Female: 0	Female: 0
Alanine aminotransferase, IU/L	NA	< 15: 0	< 15: 0
		15–44: 1	15–44: 1
		≥ 45: 2	≥ 45: 2
HBeAg	NA	Positive: 2	Positive: 2
		Negative: 0	Negative: 0
HBV DNA, log copies/mL	NA	< 4 log: 0	NA
		4–5 log: 3	
		5–6 log: 5	
		≥ 6 log: 4	
Platelet count, /mm3	≥ 200,000: 0	NA	NA
	100,000–199,999: 6		
	< 100,000: 9		
Liver stiffness, kPa	NA	≤8 kPa: 0	< 8 kPa: 0
		8.1–12 kPa: 8	8–13 kPa: 2
		> 12 kPa: 14	> 13 kPa: 4

NA, not applicable; HBeAg, hepatitis B e antigen; kPa, kilopascal.

Supplementary Table 2: Comparison of the prognostic accuracy of risk prediction models for LRE development

Study groups	At 3 years			At 5 years			At 7 years		
Prediction models	AUC	95% CI	*P* value[1]	AUC	95% CI	*P* value[1]	AUC	95% CI	*P* value[1]
All (*n* = 1,241)									
PAGE-B	0.704**	0.631–0.777	0.048	0.721**	0.664–0.778	0.602	0.704**	0.650–0.758	0.128
REACH-B	-	-	-	-	-	-	-	-	-
LSM-HCC	0.777**	0.706–0.848	0.798	0.735**	0.675–0.796	0.809	0.742**	0.688–0.796	0.465
mREACH-B	0.782**	0.711–0.853	-	0.739**	0.681–0.798	-	0.753**	0.702–0.803	-
With normal ALT (*n* = 707)									
PAGE-B	0.738*	0.655–0.820	0.388	0.708**	0.636–0.781	0.727	0.685**	0.607–0.763	0.436
REACH-B	-	-	-	-	-	-	-	-	-
LSM-HCC	0.779**	0.693–0.864	0.684	0.739**	0.663–0.814	0.630	0.719**	0.647–0.792	0.888
mREACH-B	0.792**	0.699–0.885	-	0.726**	0.648–0.805	-	0.723**	0.653–0.793	-
With high ALT (*n* = 534)									
PAGE-B	0.680*	0.564–0.796	0.027	0.739**	0.649–0.828	0.684	0.733**	0.658–0.807	0.164
REACH-B	-	-	-	-	-	-	-	-	-
LSM-HCC	0.776**	0.663–0.889	0.706	0.737**	0.641–0.834	0.363	0.768**	0.689–0.846	0.352
mREACH-B	0.768**	0.659–0.876	-	0.756**	0.671–0.841	-	0.786**	0.717–0.855	-
Without cirrhosis (*n* = 940)									
PAGE-B	0.673	0.537–0.810	0.422	0.729**	0.642–0.817	0.435	0.670*	0.572–0.767	0.879
REACH-B	0.619	0.494–0.745	0.203	0.626*	0.519–0.733	0.481	0.600	0.494–0.705	0.222
LSM-HCC	0.789*	0.697–0.880	0.256	0.709*	0.615–0.813	0.299	0.701**	0.605–0.796	0.507
mREACH-B	0.734*	0.602–0.883	-	0.674*	0.570–0.779	-	0.680*	0.591–0.769	-
With cirrhosis (*n* = 301)									
PAGE-B	0.592	0.481–0.703	0.210	0.607*	0.514–0.700	0.536	0.607*	0.524–0.689	0.244
REACH-B	-	-	-	-	-	-	-	-	-
LSM-HCC	0.663*	0.549–0.777	0.722	0.631*	0.538–0.725	0.706	0.645*	0.563–0.728	0.391
mREACH-B	0.674*	0.568–0.780	-	0.641*	0.556–0.726	0.706	0.666**	0.590–0.741	-

[1]*P* value indicates the comparison between mREACH-B and other prediction models. AUC* and ** indicate *P* value of < 0.05 and < 0.001, respectively.
LRE, liver-related event; AUC, area under curve; CI, confidence interval; ALT, alanine aminotransferase.
